# Analysis of Copy Number Variation in Alzheimer’s Disease in a Cohort of Clinically Characterized and Neuropathologically Verified Individuals

**DOI:** 10.1371/journal.pone.0050640

**Published:** 2012-12-05

**Authors:** Shanker Swaminathan, Matthew J. Huentelman, Jason J. Corneveaux, Amanda J. Myers, Kelley M. Faber, Tatiana Foroud, Richard Mayeux, Li Shen, Sungeun Kim, Mari Turk, John Hardy, Eric M. Reiman, Andrew J. Saykin, the Alzheimer's Disease Neuroimaging Initiative (ADNI) and the NIA-LOAD/NCRAD Family Study Group

**Affiliations:** 1 Center for Neuroimaging, Department of Radiology and Imaging Sciences, Indiana University School of Medicine, Indianapolis, Indiana, United States of America; 2 Department of Medical and Molecular Genetics, Indiana University School of Medicine, Indianapolis, Indiana, United States of America; 3 Neurogenomics Division, The Translational Genomics Research Institute (TGen), Phoenix, Arizona, United States of America; 4 The Arizona Alzheimer's Consortium, Phoenix, Arizona, United States of America; 5 Departments of Psychiatry and Behavioral Sciences, and Human Genetics and Genomics, University of Miami, Miller School of Medicine, Miami, Florida, United States of America; 6 Johnnie B. Byrd Sr. Alzheimer's Center and Research Institute, Tampa, Florida, United States of America; 7 Center for Computational Biology and Bioinformatics, Indiana University School of Medicine, Indianapolis, Indiana, United States of America; 8 The Gertrude H. Sergievsky Center, The Taub Institute for Research on Alzheimer's Disease and the Aging Brain, and the Department of Neurology, Columbia University College of Physicians and Surgeons, New York, New York, United States of America; 9 Department of Molecular Neuroscience and Reta Lila Research Laboratories, University College London Institute of Neurology, London, United Kingdom; 10 Banner Alzheimer’s Institute, Phoenix, Arizona, United States of America; Vanderbilt University, United States of America

## Abstract

Copy number variations (CNVs) are genomic regions that have added (duplications) or deleted (deletions) genetic material. They may overlap genes affecting their function and have been shown to be associated with disease. We previously investigated the role of CNVs in late-onset Alzheimer's disease (AD) and mild cognitive impairment using Alzheimer’s Disease Neuroimaging Initiative (ADNI) and National Institute of Aging-Late Onset AD/National Cell Repository for AD (NIA-LOAD/NCRAD) Family Study participants, and identified a number of genes overlapped by CNV calls. To confirm the findings and identify other potential candidate regions, we analyzed array data from a unique cohort of 1617 Caucasian participants (1022 AD cases and 595 controls) who were clinically characterized and whose diagnosis was neuropathologically verified. All DNA samples were extracted from brain tissue. CNV calls were generated and subjected to quality control (QC). 728 cases and 438 controls who passed all QC measures were included in case/control association analyses including candidate gene and genome-wide approaches. Rates of deletions and duplications did not significantly differ between cases and controls. Case-control association identified a number of previously reported regions (*CHRFAM7A*, *RELN* and *DOPEY2*) as well as a new gene (*HLA-DRA*). Meta-analysis of *CHRFAM7A* indicated a significant association of the gene with AD and/or MCI risk (*P* = 0.006, odds ratio = 3.986 (95% confidence interval 1.490–10.667)). A novel *APP* gene duplication was observed in one case sample. Further investigation of the identified genes in independent and larger samples is warranted.

## Introduction

Alzheimer’s disease (AD) is the most common form of dementia characterized by loss of memory and other cognitive abilities, severe enough to disrupt daily life activities. An estimated 5.4 million Americans have AD, the sixth leading cause of death across all ages in the United States [1]. No treatments at present can slow or halt its progression. Amnestic mild cognitive impairment (MCI) is a clinical condition in which a person has memory problems not normal for his/her age, but not severe enough to interfere significantly with daily functioning. Approximately 14–18% of individuals aged 70 years and older have MCI, and every year 10–15% of these individuals will likely progress to dementia, particularly AD [2].

Genetic factors play a key role in AD development accounting for approximately 58–79% of the phenotypic variation [3]. Mutations in *APP*, *PSEN1* and *PSEN2* primarily cause early-onset AD (age at onset<60 or 65 years) [4]. The leading genetic risk factor for the more common late-onset AD (age at onset>60 or 65 years) is the *APOE* ε4 allele. Large case-control genome-wide association studies (GWASs) have identified and replicated other AD risk loci including: *CLU*, *CR1*, *PICALM*, *BIN1*, *EXOC3L2*, *MTHFD1L*, *MS4A4A/MS4A6E*, *CD2AP*, *CD33*, *ABCA7* and *CUGBP2* [5]–[12]. However it is estimated that the *APOE* ε4 allele accounts for approximately 20% and the non-*APOE* loci cumulatively account for as much as 35% of the AD risk [10], [13]. A recent study observed only a marginal joint effect of known loci on memory independent from *APOE* [14]. The combined loci provided minimal improvement of prediction of AD beyond age, sex and *APOE*. Thus the loci do not explain all the genetic variation associated with AD, and other forms of genetic variation such as copy number variations (CNVs) may play a role.

CNVs are deoxyribonucleic acid (DNA) regions (one kilobase (kb) to several megabases (Mb) in size) that have differences in copy number. These can result in the addition (copy number gains or duplications) or loss (copy number losses or deletions) of genetic material. CNVs often encompass a single gene or multiple genes and may affect their function [15]. The role of CNVs in late-onset AD has been investigated in prior studies [16]–[19]. Previously, we analyzed the role of CNVs in AD and MCI using data from participants in the Alzheimer’s Disease Neuroimaging Initiative (ADNI) study [20] and the National Institute of Aging-Late Onset AD/National Cell Repository for AD (NIA-LOAD/NCRAD) Family Study [21]. For both studies, DNA extracted either from peripheral blood or brain tissue were used. Case/control association analyses including candidate gene and genome-wide approaches were performed to determine genes overlapped by CNVs only in cases (AD and/or MCI) but not controls. A number of genes were identified in the two studies including *ATXN1*, *CHRFAM7A*, *CSMD1*, *DOPEY2*, *ERBB4*, *GSTT1*, *HLA-DPB1*, *HNRNPCL1*, *IMMP2L*, *NRXN1*, *RELN* and *SLC35F2*.

The aim of the present report is to analyze the role of CNVs in AD using data from a unique cohort of clinically characterized and neuropathologically defined cases (AD) and controls (TGen cohort) [22]. All DNA samples were extracted from brain tissue. Case/control association analyses similar to the two previous studies were performed to determine the CNV burden in cases relative to controls and genes overlapped by CNVs detected in cases but not controls. Here we report analyses identifying a number of previously reported as well as new CNV regions.

## Materials and Methods

### Samples

The TGen cohort included samples extracted from brain tissue of 1617 Caucasian individuals (1022 AD cases and 595 controls). Recruitment information for the participants has been previously described [22]. Briefly, the United States cohort was obtained from 21 National Institute on Aging-supported Alzheimer's Disease Center brain banks and from the Miami Brain Bank [23], [24]. Cohorts from other brain banks in the United States, United Kingdom, and the Netherlands were obtained similar to the original United States cohort. Genome-wide genotyping for all samples was performed using the Affymetrix Genome-Wide Human SNP 6.0 Array (Santa Clara, California, United States of America) as previously described [22]. *APOE* genotyping was done using Crook *et al.*'s method [25] or using a fluorescence-based allele-specific polymerase chain reaction (PCR), also called PCR Amplification of Specific Alleles, on array tape [26] by PreventionGenetics (Marshfield, Wisconsin, United States of America).

The ADNI data used in the preparation of the present report were obtained from the Alzheimer's Disease Neuroimaging Initiative (ADNI) database (http://adni.loni.ucla.edu/). ADNI's primary goal is to test whether imaging markers, genetic markers, other biological markers, and clinical and neuropsychological assessments can be combined to measure progression of MCI and early AD. More information on ADNI can be found on http://www.adni-info.org/. The Illumina Human610-Quad BeadChip (San Diego, California, United States of America) was used to perform genome-wide genotyping of the ADNI sample as previously described [20], [27]. The *APOE* polymorphisms (rs429358 and rs7412) were genotyped separately.

The NIA-LOAD/NCRAD Family Study data used in the present report were obtained from the "NIA-Late Onset Alzheimer's Disease and National Cell Repository for Alzheimer's Disease Family Study: Genome-Wide Association Study for Susceptibility Loci" dataset (dbGaP Study Accession: phs000168.v1.p1, Project #2026) on the database of Genotypes and Phenotypes (http://www.ncbi.nlm.nih.gov/projects/gap/cgi-bin/study.cgi?study_id=phs000168.v1.p1) website. Recruitment information for NIA-LOAD Family Study and NCRAD participants has been previously described [12]. Genome-wide genotyping for all samples was performed using the Illumina Human610-Quad BeadChip at the Center for Inherited Disease Research (Baltimore, Maryland, United States of America). The *APOE* polymorphisms (rs429358 and rs7412) were genotyped at PreventionGenetics.

### Ethics Statement

De-identification of samples in the TGen cohort was done before receipt, and the study met human studies institutional review board and the Health Insurance Portability and Accountability Act of 1996 regulations. The present work is declared not human-subjects research and is institutional review board exempt under regulation 45 CFR 46. The ADNI study was approved by institutional review boards of all participating institutions and written informed consent was obtained from all participants or authorized representatives. All individuals in the NIA-LOAD/NCRAD Family Study were recruited after providing informed consent and with approval by the relevant institutional review boards. The study was conducted according to the principles in the Declaration of Helsinki.

### Generation of CNV Calls and Quality Control

CNV calls were generated for the 1617 TGen samples using PennCNV (2011Jun16 version; http://www.openbioinformatics.org/penncnv/), a Hidden Markov model based program [28]. The PennCNV-Affy protocol (http://www.openbioinformatics.org/penncnv/penncnv_tutorial_affy_gw6.html) for the Affymetrix Genome-Wide Human SNP 6.0 Array was first performed to transform raw CEL files into a signal intensity file containing the Log R Ratio (LRR) and B Allele Frequency (BAF) values used by PennCNV to generate CNV calls. The Hidden Markov model “affygw6.hmm”, population frequency of B allele “affygw6.hg18.pfb” and gcmodel “affygw6.hg18.gcmodel” files were used. Extensive quality control (QC) was performed on all samples. A genomic wave adjustment procedure using PennCNV’s gcmodel file was applied as samples that have below optimal genomic wave QC values can be considered unreliable [29]. Frequency distribution plots of the number of CNV calls, LRR standard deviation (SD), BAF Drift and Waviness Factor (WF) were made. A sample was excluded if at least one of the above measures for the sample was greater than 90^th^ percentile of the frequency distribution, i.e. the sample had >56 CNV calls, LRR SD>0.38, BAF Drift>0.01 or WF>0.02.

Due to complications of hemizygosity in males and X-chromosome inactivation in females, analyses were restricted to autosomes. To ensure we were including only high-confidence CNVs in the analysis, CNVs for which the difference of the log likelihood of the most likely copy number state and less likely copy number state was <10, CNVs called based on data <10 SNPs, and CNVs that had >50% overlap with centromeric, telomeric, and immunoglobulin regions as defined in Need *et al.* [30] were excluded. CNV calls were not filtered for size because both large and small variants could be of potential significance. A case sample observed to have a very large (∼8.4 Mb) deletion on chromosome 19, and a control sample observed to have a very large (∼22.4 Mb) duplication on chromosome 1, were excluded from the analyses as they may be possible outliers. The ∼8.4 Mb deletion on chromosome 19 encompassed both sides of the centromere, but did not overlap any RefSeq or UCSC Genes according to the UCSC Genome Browser [31] (http://genome.ucsc.edu/). 1166 samples (728 cases, 438 controls) with 31045 CNV calls remained after all QC measures and were entered into case/control association analyses.

### Case/control Association Analyses

Case/control analyses using permutation-based tests of association in the TGen study were performed similar to the ADNI [20] and NIA-LOAD/NCRAD Family [21] studies. PLINK v1.07 [32] (http://pngu.mgh.harvard.edu/~purcell/plink/) was used to investigate CNV call differences between cases (AD) and controls. Two approaches were used: a candidate gene approach including 317 AD genes identified from the AlzGene database (Updated 5 January 2011) (http://www.alzgene.org/) as having a positive association with AD in at least one study, and a genome-wide approach using 17938 genes from PLINK’s gene list (hg18 coordinates). The AlzGene database is a publicly available online resource that provides a comprehensive and regularly updated catalog of genetic case/control and family association studies in AD [33]. In both approaches, CNV segments either partially or completely overlapping genes were analyzed. The analyses included both deletions and duplications. 50000 null permutations were performed to generate one-sided empirical *P* values testing genes overlapped by CNV calls in more cases than controls. The analyses focused on genes overlapped by CNV calls in cases, but not in controls, to identify genes that may play a role in AD susceptibility. 317 genes were considered in the candidate gene approach and 17938 genes were considered in the genome-wide approach. Genes that achieved *P*<0.05 (one-sided) were considered significant.

### Meta-analysis

**Figure 1:**
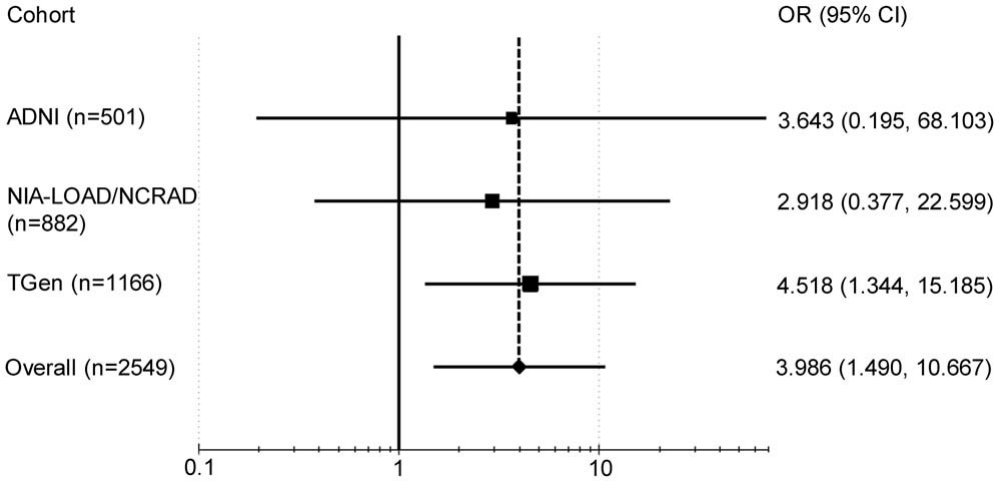
Forest plot of the *CHRFAM7A* gene. The plot represents the meta-analysis of the *CHRFAM7A* gene using results from the Alzheimer’s Disease Neuroimaging Initiative (ADNI) study, the National Institute of Aging-Late Onset AD/National Cell Repository for AD (NIA-LOAD/NCRAD) Family Study and the TGen study. The odds ratio (OR) and 95% confidence interval (CI) for the odds ratio for each study are represented by black squares and horizontal lines. The summary odds ratio is depicted as a black diamond.

**Figure 2:**
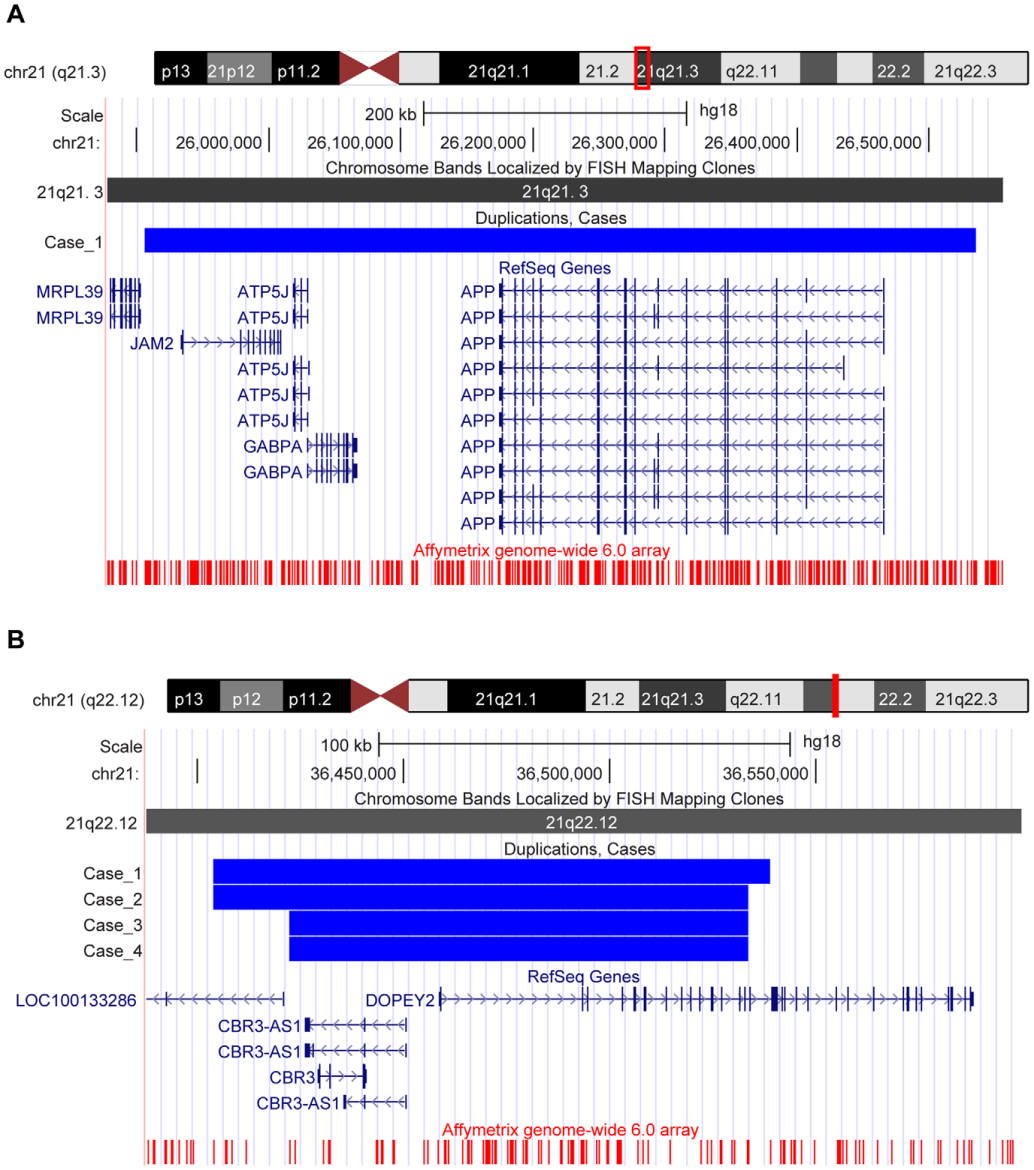
Duplications overlapping the candidate genes *APP* and *DOPEY2* in samples of the TGen cohort. Representative UCSC Genome Browser (March 2006 (NCBI36/hg18) assembly) plots of duplications overlapping the candidate genes: (A) *APP* and (B) *DOPEY2*, in samples of the TGen cohort. The chromosomal location of the gene and probes on the Affymetrix Genomewide 6.0 array are shown. The region with the duplication for each sample relative to the gene is represented by a blue rectangle.

**Figure 3:**
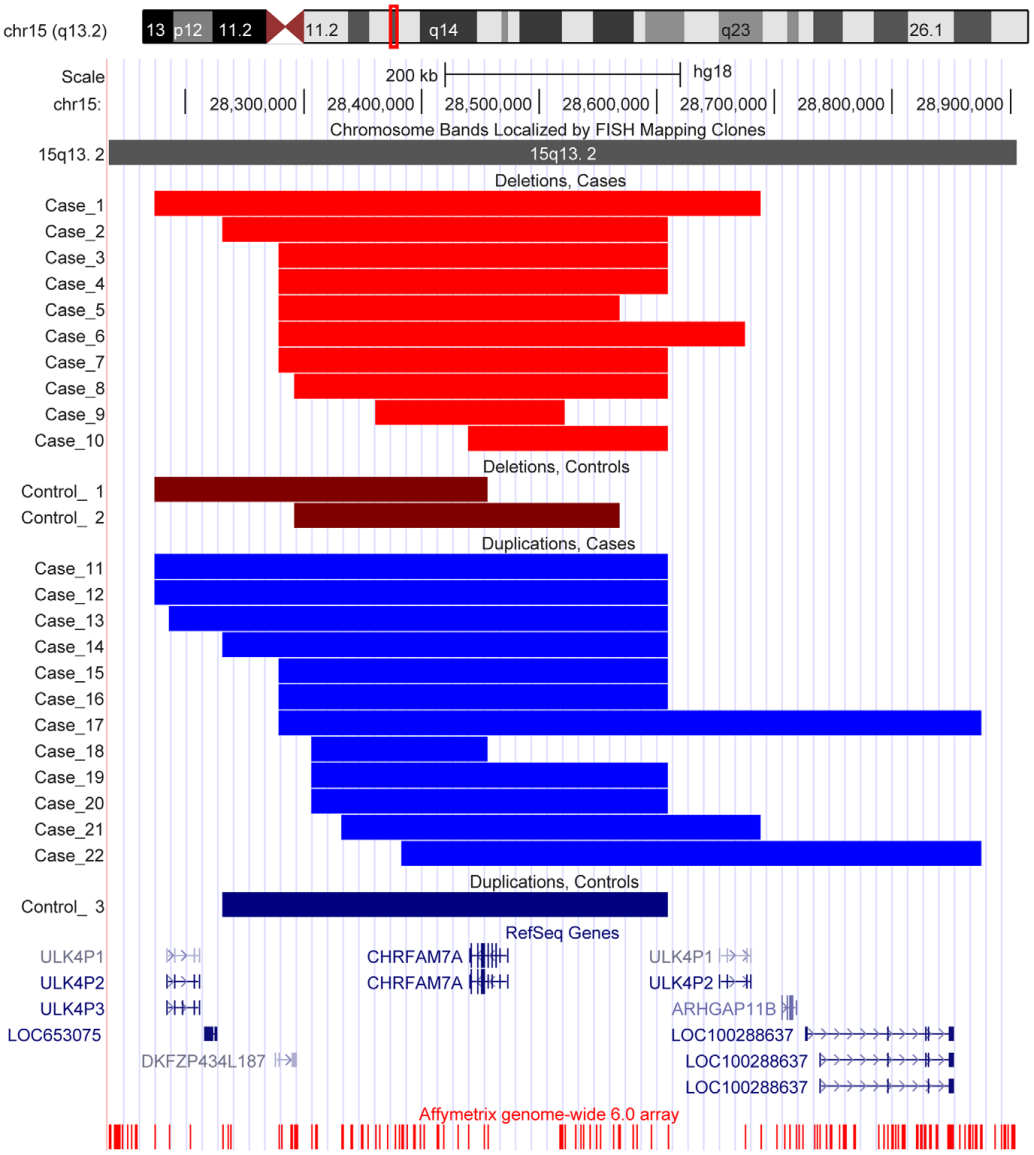
Deletions and duplications overlapping the *CHRFAM7A* gene in samples of the TGen cohort. Representative UCSC Genome Browser (March 2006 (NCBI36/hg18) assembly) plots of deletions and duplications overlapping the *CHRFAM7A* gene in samples of the TGen cohort. The chromosomal location of the gene and probes on the Affymetrix Genomewide 6.0 array are shown. The region with the deletion for each sample relative to the gene is highlighted by a red rectangle and the region with the duplication for each sample relative to the gene is highlighted by a blue rectangle.

We performed a meta-analysis for the *CHRFAM7A* gene using results from the ADNI, NIA-LOAD/NCRAD Family and TGen studies to determine differences in frequency of CNV calls overlapping the gene between cases (AD and/or MCI) and controls. A fixed-effects model was run and a summary odds ratio (OR) was calculated using the Mantel Haenszel method. MetaAnalyst Beta 3.13 [34] (http://tuftscaes.org/meta_analyst/index.html) and Comprehensive Meta-Analysis Version 2 [35] were used for the meta-analysis and generation of the forest plot (Figure 1). The UCSC Genome Browser [31] (http://genome.ucsc.edu/) (March 2006 (NCBI36/hg18) assembly) was used to create representative plots of the CNV calls (Figures 2 and 3). The Genome Browser track for the Affymetrix Genomewide 6.0 array was obtained from the PennCNV website (http://www.openbioinformatics.org/penncnv/penncnv_download.html).

## Results

### Sample Demographics and CNV Call Characteristics

The sample demographics and CNV call characteristics of the 728 cases and 438 controls who passed all QC measures are shown in Tables 1 and 2. Significant (*P*<0.05; two-sided) differences in gender, absence or presence of the *APOE* ε4 allele, age at death, Braak stage and the Consortium to Establish a Registry for Alzheimer's Disease (CERAD) score were observed between cases and controls. 31045 CNV calls (24188 deletions and 6857 duplications) were observed with an average of 45 SNPs per CNV call and an average CNV call length of 64.76 kb. A higher CNV call rate and a lower average CNV call size were observed in deletions compared to duplications. Rates of deletions and duplications did not significantly differ between cases and controls. There were no significant differences in the rates of deletions and rates of duplications when males and females were analyzed separately (data not shown). A large proportion of deletions and duplications were found in the 0.1–0.5 Mb size range (Table 3).

**Table 1:** Sample demographics in the TGen cohort.

	Cases	Controls	*P* (two-sided)
Number of samples	728	438	−
Gender (Males/Females)	180/548	274/164	<0.001
*APOE* group (ε4 negative/ε4 positive)	93/173	138/45	<0.001
Age at onset	72.84±6.14 (n = 60)	−	−
Age at death	82.46±7.58 (n = 266)	80.80±9.17 (n = 183)	0.037
Braak stage	5.09±0.83 (n = 168)	1.62±0.76 (n = 96)	<0.001
CERAD^a^ score	2.31±0.84 (n = 26)	0.83±0.64 (n = 47)	<0.001

a CERAD = The Consortium to Establish a Registry for Alzheimer’s Disease.

**Table 2:** Characteristics of copy number variation calls from samples in the TGen cohort.

	Cases (n = 728)	Controls (n = 438)
**Deletions**		
Number of copy number variation calls	15177	9011
Rate per sample	20.85	20.57
Average size (kilobase)	45.34	46.75
**Duplications**		
Number of copy number variation calls	4334	2523
Rate per sample	5.95	5.76
Average size (kilobase)	140.8	131.1

**Table 3:** TGen samples grouped by copy number variation call size.

Call size (Megabase)	Cases (n = 728)		Controls (n = 438)	
	**Deletions n (%)**	**Duplications n (%)**	**Deletions n (%)**	**Duplications n (%)**
0.1–0.5	647 (88.87)	625 (85.85)	387 (88.36)	368 (84.02)
0.5–1.0	95 (13.05)	164 (22.53)	61 (13.93)	101 (23.06)
1.0–1.5	10 (1.37)	25 (3.43)	7 (1.60)	12 (2.74)
1.5–2.0	4 (0.55)	7 (0.96)	0 (0.00)	2 (0.46)
>2.0	2 (0.27)	11 (1.51)	5 (1.14)	1 (0.23)

### Case/control Association Analyses

The candidate gene approach identified 32 of the 317 genes tested (10.09%) and the genome-wide approach identified 939 of the 17938 genes tested (5.23%) to be overlapped by CNV calls only in cases (AD) but not controls in the TGen study. A significant (*P* = 0.0003; Fisher’s exact test; two-sided) enrichment of the candidate genes relative to the genome was observed.

#### Candidate gene approach

We identified 32 candidate genes in the TGen study overlapped by CNV calls from at least one case (AD) but no controls (Table 4). Representative plots of two genes (*APP* and *DOPEY2*) are shown in Figure 2. The *HLA-DRA* gene was overlapped by deletions in nine cases (uncorrected *P* = 0.0140; one-sided). This gene was also found to be overlapped by deletions in two controls in the ADNI study. Two genes (*RELN* overlapped by deletions in two cases and *DOPEY2* overlapped by duplications in four cases) identified in this study were also reported from only cases (AD and/or MCI) in the ADNI and NIA-LOAD/NCRAD Family studies. One AD sample (*APOE* ε2/ε3 genotype, age at death = 67) had a novel *APP* gene duplication supported by 443 sequential SNP and CNV probes. The *CHRFAM7A* gene reported in the ADNI and NIA-LOAD/NCRAD Family studies was overlapped by deletions in 10 cases and two controls, and duplications in 12 cases and one control (corrected *P* = 0.0198; one-sided) in this study (Figure 3).

**Table 4:** Genes overlapped by copy number variation calls from at least one case and no control samples in the TGen cohort using the candidate gene approach.

Chromosome	Region	Start (base pair)	End (base pair)	Number of cases
1	*FAM63A*	149234172	149245957	1
1	*SOAT1*	177529639	177591076	1
1	*AGT*	228904891	228916959	1^a^
2	*LHCGR*	48767416	48836384	4^a^
6	*HLA-G*	29902734	29906878	1
6	*HLA-A*	30018309	30021633	2
6	*HLA-E*	30565249	30569072	3^b^
6	*BAT1*	31605974	31618204	1^a^
6	*HLA-DRA*	32515624	32520802	9^b,c,d^
6	*HLA-DQB1*	32735634	32742444	1^c^
7	*MAGI2*	77484309	78920826	1
7	*CD36*	80069439	80146529	1
7	*RELN*	102899472	103417198	2
8	*NAT2*	18293034	18303003	1
10	*ALDH18A1*	97355675	97406557	1
10	*EBF3*	131523536	131652081	1
11	*PICALM*	85346132	85457756	1
12	*C12orf41*	47333261	47362302	3
12	*ALDH2*	110688728	110732167	2
14	*PSEN1*	72672931	72756862	1
14	*NGB*	76801586	76807408	1^d^
17	*SERPINF2*	1592879	1605309	1
17	*MYH13*	10144907	10217047	2^e^
17	*MYH8*	10234366	10265992	1^e^
17	*MAPT*	41327543	41461546	1
19	*GALP*	61379200	61388956	1
21	*APP*	26174731	26465003	1
21	*DOPEY2*	36458708	36588442	4
21	*CBS*	43346369	43369493	1
21	*S100B*	46842958	46849463	1
22	*COMT*	18309308	18336530	1
22	*BCR*	21852551	21990224	1

a A case sample had copy number variation calls overlapping the *AGT*, *LHCGR* and *BAT1* genes.

b,c,d Three different case samples had copy number variation calls overlapping the *HLA-DRA* and *HLA-E*, *HLA-DRA* and *HLA-DQB1*, and *HLA-DRA* and *NGB* genes respectively.

e A different case sample had copy number variation calls overlapping the *MYH13* and *MYH8* genes.

#### Genome-wide approach

We also identified 939 genes across the genome overlapped by CNV calls only in cases (AD) but not controls in the TGen study. Genes overlapped by CNV calls from at least four cases but not controls in the TGen study are shown in Table 5. The *HLA-DRA* gene overlapped by deletions in nine cases in the TGen study and identified in the candidate gene approach was also found from this approach (uncorrected *P* = 0.0144; one-sided). The *CHRFAM7A* gene reported in the ADNI and NIA-LOAD/NCRAD Family Studies and mentioned in the candidate gene approach had an uncorrected *P* = 0.0046; one-sided. Deletions in 10 cases and two controls, and duplications in 12 cases and one control, overlapped the *CHRFAM7A* gene in the TGen study (Figure 3). Other genes reported in the ADNI and NIA-LOAD/NCRAD Family studies were overlapped by CNV calls in the TGen study although they did not reach significance at uncorrected *P*<0.05. These include *CSMD1* (deletions in 65 cases and 32 controls, duplications in one case and one control), *ERBB4* (deletions in 71 cases and 35 controls, duplication in one control), *HNRNPCL1* (deletions in 19 cases and eight controls, duplications in one case and two controls), *IMMP2L* (deletions in six cases and five controls, duplication in one control), *NRXN1* (deletions in two cases and three controls), and *SLC35F2* (duplications in two cases and one control). Deletions in three cases and three controls, and duplications in two cases and three controls in the TGen study, overlapped the *CHRNA7* gene identified in a previous study [16]. No CNV calls were found in the TGen study overlapping other previously reported regions including *CR1* [17], 14q11.2 [18] and 15q11.2 [19].

**Table 5:** Genes overlapped by copy number variation calls in at least four case samples and no control samples in the TGen cohort using the genome-wide approach.

Chromosome	Region	Start (base pair)	End (base pair)	Number of cases
1	*LCE1D*	151035850	151037281	6
2	*GTF2A1L*	48698451	48813790	4^a^
2	*LHCGR*	48767416	48836384	4^a^
4	*TMPRSS11E2*	69465107	69515259	6
6	*HLA-DRA*	32515624	32520802	9^b^
7	*RP9*	33100934	33115527	4
11	*OR52B4*	4345156	4346101	4^c^
11	*GRM5*	87880625	88420838	4
11	*ST3GAL4*	125731305	125789743	4^c^
21	*CBR3*	36429132	36440730	4^d^
21	*DOPEY2*	36458708	36588442	4^d^
22	*DGCR2*	17403794	17489967	5^b^

a Four case samples had copy number variation calls overlapping the *GTF2A1L* and *LHCGR* genes.

b A different case sample had copy number variation calls overlapping the *HLA-DRA* and *DGCR2* genes.

c A different case sample had copy number variation calls overlapping the *OR52B4* and *ST3GAL4* genes.

d Four different case samples had copy number variation calls overlapping the *CBR3* and *DOPEY2* genes.

### Meta-analysis

A meta-analysis for the *CHRFAM7A* gene was performed using findings from the ADNI, NIA-LOAD/NCRAD Family and TGen studies (Table 6 and Figure 1). Overall, the gene was overlapped by CNV calls in 38 of 1797 (2.115%) cases (AD and/or MCI) and four of 752 (0.532%) controls. A significant association was observed for the gene with AD and/or MCI risk (*P* = 0.006, summary OR = 3.986 (95% confidence interval 1.490–10.667)).

**Table 6:** Meta-analysis of the *CHRFAM7A* gene.

Cohort	ADNI (n = 501)	NIA-LOAD/NCRAD (n = 882)	TGen (n = 1166)	Overall (n = 2549)
**Cases**				
Number of samples	358	711	728	1797
CNV^a^ overlapping gene (n)	4	12	22	38
No CNV^a^ overlapping gene (n)	354	699	706	1759
**Controls**				
Number of samples	143	171	438	752
CNV^a^ overlapping gene (n)	0	1	3	4
No CNV^a^ overlapping gene (n)	143	170	435	748
Odds ratio	3.643	2.918	4.518	3.986
95% confidence interval	0.195–68.103	0.377–22.599	1.344–15.185	1.490–10.667
*P*	0.387	0.305	0.015	0.006

a CNV = copy number variation.

## Discussion

The present report represents an initial CNV analysis in the TGen cohort, a unique cohort of clinically characterized and neuropathologically verified individuals. After extensive QC, case (AD)/control association analyses using candidate gene and genome-wide approaches were performed. Genes enriched in cases relative to controls were determined, suggesting possible involvement of these genes in AD susceptibility.

Rates of deletions and duplications did not significantly differ between cases and controls. This is different from findings in the ADNI and NIA-LOAD/NCRAD Family studies and could be due to different participant selection criteria, random sampling variation, different QC criteria, and that the NIA-LOAD/NCRAD Family Study and TGen study analyses included only AD and control samples, whereas the ADNI study analyses included MCI samples in addition to AD and control samples.

The candidate gene approach revealed a number of interesting genes (Table 4 and Figure 2). The *HLA-DRA* (major histocompatibility complex, class II, DR alpha) gene on chromosome 6 is a human leukocyte antigen (HLA) class II alpha chain paralogue which plays an important role in the immune system by presenting peptides derived from extracellular proteins. Variants in *HLA-DRA* have been associated with Parkinson disease [36], [37] and multiple sclerosis [38], [39], but not with AD. Other *HLA* alleles however have been investigated for a possible role in AD [40]–[43].

The *CHRFAM7A* (*CHRNA7* (cholinergic receptor, nicotinic, alpha 7, exons 5–10) and *FAM7A* (family with sequence similarity 7A, exons A–E) fusion) gene (Figure 3) is located on chromosome 15. It is formed as a hybrid of a partially duplicated *CHRNA7* gene and the *FAM7A* gene [44], [45]. It is highly polymorphic and individuals with and without this gene have been identified. A 2-bp deletion polymorphism at position 497–498 in exon 6 of this gene has been observed to be significantly over-represented in participants with AD, dementia with Lewy bodies and Pick’s disease compared to controls [46].

Although *CHRFAM7A* is transcribed, its translation and possible function of the resulting protein is uncertain. The gene is expressed in the hippocampus, a brain region known to be first affected in AD. Recently, it has been suggested to possibly modulate α7 subunit receptor-mediated synaptic transmission and cholinergic anti-inflammatory response [47]. It may also be a dominant negative modulator of *CHRNA7* function and important for receptor regulation in humans [48]. Duplications overlapping the *CHRNA7* gene and an approximately 300 kb region upstream of the gene were identified in a previous study investigating the role of CNVs in AD [16]. A meta-analysis using the *CHRFAM7A* gene results from the ADNI, NIA-LOAD/NCRAD Family and TGen studies indicated a significant association of the gene with AD and/or MCI risk at an OR of 3.986. This association is striking relative to known AD markers and is similar to the OR for one copy of the *APOE* ε4 allele. However, unlike the *APOE* ε4 allele which is a change in a single base within the gene, CNV calls overlapping the *CHRFAM7A* gene involves more than one base and often a large region with less than perfect definition of boundaries given available methodology. In addition, in a CNV analysis, a gene may be overlapped by deletions or duplications which may have different effects on the protein. Nonetheless, the strength of CNV signals overlapping the *CHRFAM7A* gene in the present three samples warrants further investigation and replication in additional independent samples.

Two genes (*RELN* and *DOPEY2*) in the three studies were found to be overlapped by CNV calls from cases (AD and/or MCI) but not controls. The *RELN* (reelin) gene on chromosome 7 encodes the glycoprotein reelin, which activates a signaling pathway required for proper positioning of neurons within laminated nervous system parenchyma. Gene variants have been associated with AD [49] and the protein has been observed to have increased expression in pyramidal neurons of the hippocampus in AD individuals and in cognitively intact controls with AD-associated pathology [50]. The *DOPEY2* (dopey family member 2, also known as *C21orf5*) gene located on chromosome 21 in the Down syndrome critical region is a potential Down syndrome candidate gene [51], [52]. Overexpression of the gene may be associated with the neurological phenotypes and mental retardation observed in Down syndrome patients. Although the *DOPEY2* gene was identified in the candidate gene approach in the TGen study, CNV calls overlapping this gene also completely overlapped the *CBR3* (carbonyl reductase 3) and *CBR3-AS1* (*CBR3* antisense RNA 1). The CBR3 protein, which catalyzes reduction of carbonyl compounds to their corresponding alcohols, has been found to be differentially expressed in the cerebellum of patients with atypical cases of frontotemporal lobar degeneration with fused in sarcoma-positive inclusions compared to controls [53]. Although *DOPEY2* is a known candidate, it is possible that *CBR3* and *CBR3-AS1* could be pathologic given their involvement in other neurodegenerative diseases. One AD sample (*APOE* ε2/ε3 genotype, age at death = 67) was identified with an *APP* duplication. The patient started suffering from forgetfulness at approximately 59 years and was admitted to a nursing home at approximately age 63. The patient had no reported family history of dementia. *APP* duplications have been associated with early-onset [54]–[57] but not late-onset AD, and it is possible that this patient may have had an early-onset sporadic form of AD (age at onset<60 or 65 years). Replication in independent samples and laboratory validation can help confirm the role of these genes in AD susceptibility.

The genome-wide approach revealed the candidate genes: *HLA-DRA* and *CHRFAM7A* as well as identified genes reported in the two previous studies. The *CSMD1*, *HNRNPCL1*, *IMMP2L* and *SLC35F2* genes have not been previously associated with AD. The *NRXN1* gene has been associated with autism [58], schizophrenia [59], and has been shown to have reduced expression with increasing AD severity [60]. The *ERBB4* gene may play a possible role in the progression of AD pathology [61]–[63].

It is important to note the limitations of the present report. Although the same software (PennCNV) was used in the three studies, different QC criteria were used for the selection of samples. The ADNI and NIA-LOAD/NCRAD Family study samples were genotyped on the Illumina Human610-Quad BeadChip and used similar QC criteria. The TGen study samples were genotyped on the Affymetrix Genome-Wide Human SNP 6.0 Array and thus we used a slightly different QC criteria. To our knowledge, there does not appear to be consensus on a well defined set of QC criteria for inclusion of the most appropriate samples in CNV analyses. The QC criterion applied in the present study may have been too stringent, leading to samples possibly having informative CNV data being excluded. A direct comparison of CNV calls from the three studies would be difficult as two different genotyping platforms were used. Probes from the two platforms may not correspond with each other with respect to their location. Replication in additional independent data sets and future molecular studies will help confirm the findings.

In sum, we have conducted an initial CNV analysis in samples from a cohort of clinically characterized and neuropathologically verified individuals. Rates of deletions and duplications did not significantly differ between cases and controls. Gene-based association analysis identified a number of genes including those reported in the ADNI and NIA-LOAD/NCRAD Family studies (*CHRFAM7A*, *RELN* and *DOPEY2*) as well as a new gene (*HLA-DRA*). Meta-analysis from the three studies revealed a significant association for *CHRFAM7A* with AD and/or MCI risk. Replication in independent samples will be necessary to confirm these findings. Targeted analyses of the identified regions will help determine the biological role of these variants. Overall, there appears to be some consistency of CNVs across AD cohorts and this variation holds promise for revealing novel risk factors and disease mechanisms.
